# You May Have My Help but Not Necessarily My Care: The Effect of Social Class and Empathy on Prosociality

**DOI:** 10.3389/fpsyg.2021.588017

**Published:** 2021-04-09

**Authors:** Gloria Jiménez-Moya, Bernadette Paula Luengo Kanacri, Patricio Cumsille, M. Loreto Martínez, Christian Berger

**Affiliations:** School of Psychology, Pontificia Universidad Católica de Chile, Santiago, Chile

**Keywords:** prosocial behavior, social class, empathy, helping, caring

## Abstract

Previous research has focused on the relation between social class and prosocial behavior. However, this relation is yet unclear. In this work, we shed light on this issue by considering the effect of the level of empathy and the social class of the recipient of help on two types of prosociality, namely helping and caring. In one experimental study, we found that for high-class participants, empathy had a positive effect on helping, regardless of the recipient’s social class. However, empathy had no effect for low-class participants. When it comes to caring, empathy had a positive effect for both high and low-class participants, but only when the recipient of help belonged to the same social class. This highlights that empathy by itself is not sufficient to promote cooperative relations and that the social class of the recipient of help should be taken into account to shed light on this issue.

## Introduction

In the last decades, the enactment of prosocial acts toward others and its relationship with psychological factors has become a relevant task for the understanding of current societies. Given that contemporary societies are marked by social inequality and group segregation, in this work, we focus on the relation between prosocial behavior and social class, since it is one of the most relevant social categorization that emerges from social inequality. The aim of this work is to shed light on this relation, given that previous research has shown controversial results. Furthermore, we consider two potential moderators of this relation: empathy and the social class of the recipient of help, and we focus on middle adolescence, since prosocial behavior is especially relevant for establishing positive peer relationships (Eisenberg et al., 2015). Despite the relevance of this topic, it has been predominantly studied in adult population and very little is known about the development and socialization processes involved at the individual and interpersonal level of social inequity in earlier ages. Based on this, we use an adolescent sample to shed light on how prosocial behavior extends beyond the borders of the ingroup during this critical developmental period.

Prosocial behavior refers to individuals’ tendencies to undertake voluntary actions aimed at benefiting others (Batson, 2011; see Eisenberg et al., 2015). Framed as evolutionarily functional (Warneken, 2015), the tendency to enact prosocial behaviors emerges very early in life (e.g., Warneken and Tomasello, 2007; Pettygrove et al., 2013).

Although research in prosociality converges to consider its biological and dispositional foundations (e.g., Cuadrado et al., 2016), unavoidably, the social context in which individuals develop socialization agents, and sociocultural factors among others, and shape prosocial behaviors (see Keltner et al., 2014; Malonda et al., 2019; Streit et al., 2020). In this sense, social categories that emerge from social categorization processes have proven to be a relevant factor for social responses in general (e.g., Macrae and Bodenhausen, 2001; Kawakami et al., 2002), and for prosocial behavior in particular (Guinote et al., 2015). In this work, we focus on social class categorization, given that this division is still strongly maintained in societies even though it is detrimental and enhances inequality (Piff et al., 2018). Finally, in order to inform about the roots of inequality and intergroup processes on prosocial responding, we feature a sample of middle adolescents considering their malleability to socialization processes.

## Social Class and Prosocial Behavior

We described social class as the union of both resources (power, income, education, etc.) and the position that individuals hold in the society, based on occupational prestige, preferences, tastes, etc. (e.g., Oyserman et al., 2014; Rivera and Tilcsik, 2016). Social class affects the experiences individuals live and how they see the world, thus the way in which individuals behave is also affected by this social category (e.g., Marien et al., 2010; Manstead, 2018). Consequently, the extent to which individuals care about others’ well-being is affected by their social class (e.g., Piff and Robinson, 2017).

Interestingly, the relation between prosocial behavior and social class is controversial and yet not resolved. On one hand, a negative social class effect has been described. In this line, previous research has shown that high social class individuals are less prone to help others in a wide range of situations and conceiving “helping” in different ways (see Keltner et al., 2014). For instance, Piff et al. (2010) found that low social class participants were more prosocial toward strangers when playing an economic play in a laboratory setting. Furthermore, they also showed a causal association between social class and prosocial behaviors by inducing participants to see themselves as lower than others in terms of socioeconomic status. Under this condition, participants showed greater support for charity. This effect has been related to the fact that low social class individuals endorse to a greater extent social values oriented toward egalitarianism and the well-being of others (Piff et al., 2010), and seem to be more compassionate (see van Kleef et al., 2008), what might lead them to act prosocially to a greater extent (see Piff et al., 2010; Guinote et al., 2015).

On the other hand, previous work has also pointed out that high social class individuals show more prosocial behavior than low-class people. For instance, Korndörfer et al. (2015) showed across eight studies using representative international samples that high-class individuals were more willing to make donations (see also Rajan et al., 2009), to volunteer, and to display higher rates of everyday helping behavior. Although they showed some variation in these effects as a result of including diverse moderators such us nationality or the use of different measures of social class, they found no evidence of the negative effect of social class on prosociality.

These seemingly contradictory results regarding the relation between prosociality and social class show the importance of looking at variables that may moderate this relation. In this line, in the present work, we include two aspects that might contribute to explain the opposite results reported by previous literature. Specifically, we consider the social class of the recipient of help, and the role of individual dispositions in the enactment of prosocial behaviors (i.e., empathy; Caprara et al., 2012; Eisenberg et al., 2015).

## Helping the Ingroup Fellow or the Ones in Need?

As said before, controversial results emerged when it comes to prosocial behavior and social class, but to what extent the social class of the recipient of help is relevant? Previous research has shown the tendency to help ingroup members over outgroup members, showing ingroup favoritism (see Dovidio et al., 2017). Further, this bias has been argued to be innate (Van Vugt and Park, 2010) and emerges early in life (e.g., Dunham et al., 2011; Sierksma et al., 2014). Indeed, in developmental research, less attention has been given to the ways in which social stratification and its relationship with prosocial behaviors characterize experiences of children and adolescents, in particular their decisions about helping others based on their own as well as others’ social class. However, as noticed by recent developmental research, adolescents’ attitudes toward social class contribute to several relevant developmental outcomes as peer relations, intergroup dynamics, socioemotional skills, academic achievement, and mental health (e.g., Mistry et al., 2015; Ghavami and Mistry, 2019). In particular, early and middle adolescents are developing in an incipient but forceful manner a sense of self as part of the collective. Adolescents become increasingly class-conscious throughout their development, giving value to their own social class in the context of their own identity formation (e.g., Mistry et al., 2015).

We argue that during adolescence, this ingroup effect might be also applied to the social class intergroup context, that is, social class categorization might work as an ingroup-outgroup context when it comes to prosocial behavior. In this sense, previous studies have shown that intergroup processes that take place with diverse ingroups are also applicable to the social class intergroup context. This way, individuals prefer to compare themselves to those who are similar in terms of social class (Régner and Monteil, 2007), and they distance themselves from the low low-class ingroup, when belonging to such a group becomes threatening (Kunstman et al., 2016).

Interestingly, this intergroup context (high vs. low social class) is somehow different to others, given that in this case, one of the groups is clearly more in need for help than the other, as the level of power and resources is lower for the low-class group. According to this, previous research shows that children already take into account wealth information when it comes to prosociality, believing that those who have less should be more helped (Sigelman and Waitzman, 1991; Paulus, 2014). This judgment is maintained among adults, presumably because low status individuals are less competent (Cuddy et al., 2007), thus more in need of help. In this line, recent research has shown that lower class individuals elicit greater prosociality than high-class individuals (Van Doesum et al., 2017). Therefore, based on previous research, it could be that individuals will help more those who belong to the same group or those how have fewer resources.

## Empathy and Prosocial Behavior

Research has shown the effect of empathy (described as an affective response that is alike to what other individual feels; Batson, 2011) on prosocial behavior, both directly and indirectly *via* different mechanisms (e.g., Eisenberg et al., 2010; Telle and Pfister, 2016). Empathy is a multidimensional construct that includes at least a cognitive and an affective components (usually labeled as perspective taking and empathic concern), although with some controversies around its conceptualization and measurement (see Jolliffe and Farrington, 2006; Eisenberg et al., 2010). Davis defines empathic concern as “the tendency to experience feelings of sympathy and compassion for others in need” (Davis, 1994, p. 57) and the literature is particularly clear in showing the association between the affective component of empathy with prosocial behavior (Batanova and Loukas, 2011; Jolliffe and Farrington, 2011; Streit et al., 2020). In general, empathic concern (or sympathy; Eisenberg et al., 2010), has been shown to be directly associated with prosocial responding in Western (Eisenberg et al., 2006; Carlo, 2014) and non-Western countries (Gülseven et al., 2020), whereas perspective taking appears to be a more distal precursor of prosocial behavior (Eisenberg et al., 2006). Because of its more direct and closer predictive role to prosocial behavior, in this study, we will focus on empathic concern as a key individual characteristic for prosocial intergroup helping.

In general, empathy is considered a factor that might counteract prejudices and improve positive attitudes toward people from other groups, by producing more cooperative social interactions (e.g., Batson, 2011; Boag and Carnelley, 2015). However, some researchers have pointed out that empathy might not be necessarily positive under certain conditions, and that it is prone to be influenced by social categorization processes (Tarrant et al., 2009). For instance, empathy has a positive effect when it comes to intragroup helping, but it does not affect intergroup helping (Stürmer and Siem, 2017). That is, empathy fosters individuals to help only those who are like them, and thus it may be understood as a cause of prejudice that, ironically, conducts to inequality (Bloom, 2016, 2017). Based on this evidence, we argue that empathy might be a relevant factor in shaping the relation between social class (of the helper and of the recipient of help) and prosocial behavior. We focus particularly on empathic concern as an affective dimension of empathy, considering its consistent reported associations with prosocial behavior.

## The Present Research

Our main aim is to shed light on the relation between social class and prosociality. We go beyond previous work by including three different aspects that might be critical. First, we include in the equation the social class of the recipient of help (see Van Doesum et al., 2017). Albeit we could expect that high and low social class individuals should help more those who belong to their own social group (Dovidio et al., 2017), there might be other motivations at play, for instance to help those who have less resources. Thus, we leave this as an exploratory hypothesis.

Second, we consider the role of empathy given that a large body of research has shown its effect on prosocial behavior (e.g., Eisenberg et al., 2010). When considering the interaction of empathy with social class and according to previous research (Kraus et al., 2010), we expect that empathy will have a positive effect when it comes to show prosocial behavior toward the ingroup but not toward the outgroup (Bloom, 2017; Stürmer and Siem, 2017). Further, we expect that low-class participants will show higher levels of empathy (Kraus et al., 2010). Third, we take into account the fact that prosocial behavior includes diverse behaviors (such us sharing, caring, comforting, donating, and helping; Eisenberg et al., 2015). In this line, literature distinguishes between two different types of support, namely *giving* and *doing* (Thomas and McGarty, 2017; see also Collett and Morrissey, 2007). “Giving” is related to the traditional meaning of generosity and with feelings sympathy, whereas “doing” relates to justice-oriented acts and moral outrage (Thomas and McGarty, 2017). We could argue that giving (e.g., helping, in terms of the traditional definition of prosociality) has fewer implications for the helper, and that doing (e.g., caring) implies stronger ties between the helper and the recipient of help. Thus, in this work, we distinguish between two different prosocial outcomes (included as dependent variables) that differ in terms of implications and meaning, and we explore their potential differences.

Regarding participants, we focus on middle adolescents given that prosocial tendencies are especially relevant during this developmental stage, as they support psychosocial adjustment by counteracting externalizing and internalizing problems, and reinforcing positive outcomes till adulthood (see Eisenberg et al., 2006, for a review). In addition, during adolescence, peer relations become central, and social behaviors such as prosociality constitute privileged ways to establish these interactions (Berger et al., 2015). Moreover, through the way adolescents interact with others, they determine their social positions within the peer ecology and consequently their levels of social preference and likeability (Cillessen et al., 2011). This, in turn, allows building peer norms that foster positive interpersonal bonds, or by contrast, norms that enhance discrimination and prejudice (Dijkstra and Gest, 2015; Berger and Caravita, 2016). Importantly, adolescents’ prosocial tendencies constitute a significant predictor of social cohesion and citizenship (Luengo Kanacri et al., 2016; Taylor et al., 2018; Palacios et al., 2019), and it is related to life-satisfaction and well-being already during adolescence (Ripoll-Núñez et al., 2020).

In sum, the use of an adolescent sample is a novelty of this work given that all the studies previously discussed considered adult samples.

## Materials and Methods

We ran a quasi-experimental study in the Chilean context, where segregation based on social class is very explicit and pervasive in multiple dimensions such as health system, geographic distribution, etc. (Agostini et al., 2008; Barozet et al., 2009; Sabatini et al., 2012). This inequality is reflected in the schools. In fact, Chile has one of the most segregated educational systems of the world, resulting in significantly unequal education opportunities (OECD, 2009). High-class families choose private schools for their children, whereas low-class families have to enroll their children in public schools, which, in most cases, are not able to offer a high-quality education. This context allowed us to get a real sample of high and low-class adolescents.

### Participants and Procedure

The sample included 262 students (*M_age_* = 13.7 years old, *SD* = 0.66; 144 female), who were recruited from seven different schools in Santiago (Chile). In order to invite schools that would fit our aims, we looked for schools with students belonging to the high and low social class groups. For that purpose, we used public information retrieved from the National Evaluation System of Quality of Education (SIMCE, for its name in Spanish, which has nearly 90% nationwide coverage), in particular parents’ reports. To operationalize social class, we used an estimate of students’ socioeconomic status (SES) based on the mother’s and father’s years of education, and household per capita income (see Treviño et al., 2016; see also Contreras et al., 2010; Mizala and Torche, 2012; Valenzuela et al., 2014). With this information, we created five different groups according to SES reported by the parents (from group 1-students with the lowest scores to group 5-students with the highest scores). Based on the percentage of students that each school had on each of the five groups, we categorized them into low-class (with students from groups 1 and 2), middle-class (with students predominantly from group 3), and high-class schools (with students from groups 4 and 5). According to the aim of the study, we invited to participate four low-class schools (low-class condition) and four high-class schools (high-class condition). Seven of these eight schools agreed to participate in the study (four low-class and three high-social schools), and we planned to recruit as many participants as possible, thus all the students who presented the parental informed consent signed were invited to participate. In addition, participants signed the assent, and the study was reviewed and approved by the ethics committee of the authors’ institution.

Students filled two paper and pencil questionnaires. The first one included the empathy measure (among other variables not related to this study) and took around 15 min. One month later approximately, they filled a second questionnaire comprising the dependent variables, which took around 35 min. Specifically, participants read six different scenarios that were counterbalanced (four target scenarios and two fillers) created for this study and based on the notion of social dilemmas. In each scenario, a high or low social class adolescent, according to the experimental condition (female for female participants and a male for male participants) was in a difficult situation and asked for help to a friend (also, same gender of the participant). In line with the notion of social dilemmas, it was mentioned that choosing to help the protagonist of the story implied a cost for his or her friend, but the scenario did not specify whether the friend finally helped or not the protagonist. High and low-class participants were randomly assigned to one of the conditions, resulting in a 2 (Social class of the participant: high vs. low) × 2 (Social class of the recipient: high vs. low) between participants factorial design.

We manipulated the social class of the recipient of help based on the neighborhood they lived and on their parents’ occupations. As it was mentioned, Chile is one of the most unequal countries of the world, and this is very well-reflected in the segregation of the neighborhoods in Santiago, where some areas are only occupied and used by high social class citizens, whereas others are associated with low-class citizens. Based on this, in the low-class condition, participants read that the four protagonists of the four stories lived in well-known underprivileged areas in the city and that their parents had low-status occupations (e.g., mechanic, waiter, and cleaning lady). For participants in the high-class condition, the four recipients of help in the four stories lived in recognized privileged areas and their parents had high-status occupations (e.g., lawyer, doctor, and bank manager). In both conditions, we included two filler stories in which the recipient of help was a middle-class adolescent who lived in a neighborhood associated with this social category and the parents’ occupations were ambiguous in terms of social class (e.g., teacher and supermarket manager). Given that this was a between participants design, we included these fillers that added another level of the dependent variable (middle-class) in order to avoid suspicions among participants and increase realism. These scenarios were not analyzed as they were not part of the design of this study and were included just to gain credibility. The questionnaire and scenarios are available under request.

### Measures

#### Manipulation Checks

To check the effectiveness of the manipulation, participants rated the social class of the protagonist of each story after reading each scenario and before answering the dependent variables. For this aim, we used the MacArthur Scale of subjective social status, which has been extensively used to measure subjective social status (e.g., Shaked et al., 2016; see also Adler et al., 2000; Cundiff et al., 2013). After each scenario, participants were shown the picture of a ladder and were asked to think about the protagonist of the story and their family and to place them in the ladder. Specifically, they read: “At the top of the ladder are the people who are best off in Chile – those families who have the most money, the best education, and the most regarded jobs. At the bottom are the families who are worst off – those who have the least money, the worst education, and the least regarded jobs or no job. Make a circle on the rung where you would place [name of the recipient of help] and his/her family.”

In order to check whether participants’ identified themselves according to the objective categorization, we used the same measure to check the extent to which participants categorized themselves and their families in terms of social class. Specifically, at the end of the second questionnaire, they were asked to “make circle on the rung where you would place yourself and your family.”

#### Dependent Variables

We measured two types of prosociality, namely helping and caring (Eisenberg et al., 2015), after each scenario. We framed the helping behavior as support for helping, and created six items to measure it (*α* = 0.80; e.g., “If I was [name of the friend] I would help [name of the recipient of help]”; I think [name of the friend] will finally help [name of the recipient of help]; “I believe [name of the friend]) should help [name of the recipient of help] even though she/he has to change plans”; “I think [name of the recipient of help] actually needs [name of the friend]’s help”; “I think [name of the friend] is not a good friend if he/she does not help [name of the recipient of help]”; “It is reasonable the fact that [name of the recipient of help] asked [name of the friend] for help”). We created three items to measure caring tendencies (*α* = 0.93; i.e., “I think I would invite [name of the recipient of help] to my place”; “I think I could be friend with [name of the recipient of help]”; and “I think I could have fun with [name of the recipient of help].” In order to test the validity of both measures and make sure these two variables were indeed different variables, we ran factor analyses for each of the four scenarios, including the six helping items and the three caring items. In the four cases, analyses showed a two-factor solution. The first factor included the six helping items that explained from 45.59 to 28.66% of the variance. The second factor included the three items measuring caring, and explained from 19.70 to 23.07% of the variance.

#### Empathy

We measured empathy through empathic concerns tendencies, which is one dimension of empathy and refers to the tendency to experience feelings of warmth, sympathy, and concern toward others (Davis, 1994). Prior to the manipulation, we measured empathic concern with four items selected from the Interpersonal Reactivity Index scale (IRI; Davis, 1980; *α* = 0.60; e.g., “I often have tender, concerned feelings for people less fortunate than me”). The IRI has been previously tested in Chilean population (Fernandez et al., 2011), and has been used with Chilean adolescent samples distinguishing empathic concern as an independent dimension (Berger et al., 2015).

### Analytic Strategy

We ran regression analysis to test the effect of social class of the participant, social class of the recipient of help and empathy (included as independent variables) on support for helping and caring tendencies (dependent variables). For the analyses, we dummy coded the social class of the participant (0 = low-class and 1 = high-class), and the social class of the recipient (0 = low-class and 1 = high-class); empathy was included as a means-centered continuous predictor. In order to simplify the analyses, for each dependent variable, we created great means composites by averaging items of each target scenario, given that analysis showed high reliability among the items of each dependent variable across the scenarios. Collinearity statistics for all regression analyses were within acceptable ranges (tolerance > 0.10 and variance inflation factors < 10; Cohen et al., 2003). Table 1 shows descriptive statistics and correlations between variables.

**Table 1:** Means, SDs, and correlations for the variables included in the analysis.

		Mean (SD)	1.	2.	3.
1.	Support for helping	3.13 (0.41)	-		
2.	Caring tendencies	3.46 (0.71)	0.33	-	
3.	Empathy	3.49 (0.74)	0.21	0.21	-

All correlations are *p* < 0.001.

## Results

### Manipulation Checks

We ran an ANOVA with the recipient social class as a factor on recipient social class manipulation check. Participants in the low-class condition perceived that the social class of the character in the scenarios was lower (*M* = 4.15) than participants in the high-class condition [*M* = 8.07; *F* (1, 254) = 727.28, *p* < 0.001, *η*^2^ = 0.74]. We repeated the same analyses including the social class of the participant as a factor, and results showed no significant effects of this variable (*F* < 1, *p* = 0.84).

Furthermore, we analyzed the extent to which participants identified themselves according to their objective social class. Results showed that participants from high social class schools place themselves higher in the social class ladder (*M* = 7.75), than participants form low-class schools [*M* = 5.06; *F* (1, 248) = 217.89, *p* < 0.001, *η*^2^ = 0.47]. Therefore, we can claim that the objective social class corresponds with the subjective self-categorization.

### Main Results

#### Support for Helping

**Figure 1:**
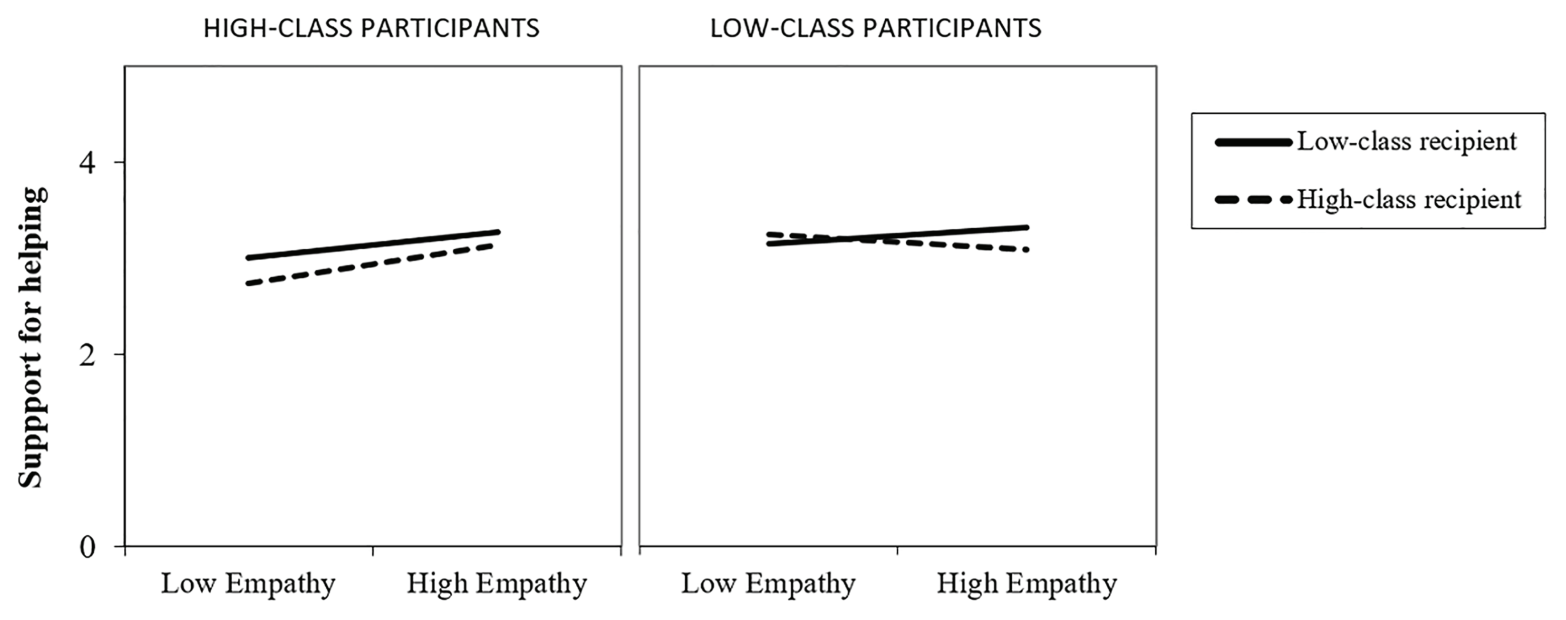
Participant’s support for helping by high and low levels of empathy, as a function of the social class of the recipient of help.

Analysis showed a significant two-way interaction between the recipient’s social class and empathy (*β* = −0.28, *p* = 0.03, *R*^2^ = 0.16, [−0.435 to −0.024]). Interestingly, this interaction was qualified by a third-way interaction, Recipient’s social class × Participant’s social class × Empathy (*β* = 0.29, *p* = 0.03, *R*^2^ = 0.16, [0.038–0.595]). Specifically, simple slopes analyses showed that for low-class participants, empathy did not affect their levels of support for helping, regardless of the social class of the recipient of help (*β* = 0.20, *p* = 0.13; *β* = −0.19, *p* = 0.15, for low-class and high-class recipients, respectively). However, for high-class participants, analysis showed a positive effect of empathy on support for helping in both conditions, that is, for the low-class recipients of help (*β* = 0.32, *p* = 0.007), and the high-class recipient of help (*β* = 0.48, *p* = 0.000; Figure 1). In conclusion, for low-class adolescents, empathy is not a relevant factor when it comes to help others, while for high-class participants, empathy does play a relevant role, as high-class adolescents who report higher levels of empathy are more likely to support the idea of helping others, regardless of the social class of the recipient of help.

#### Caring Tendencies

**Figure 2:**
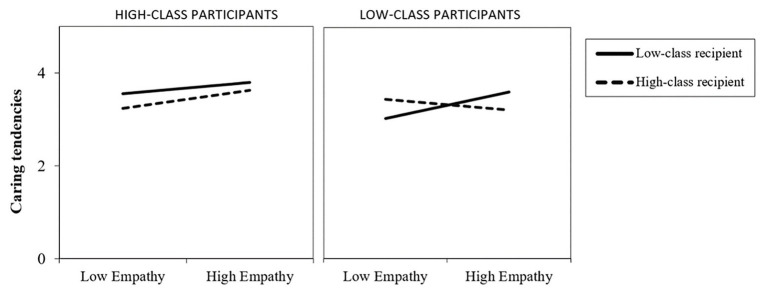
Participant’s caring tendencies by high and low levels of empathy, as a function of the social class of the recipient of help.

Analysis showed a main effect of the recipient’s social class, namely that participants showed higher caring tendencies when the recipient of help was a low social class adolescent (*β* = 1.35, *p* = 0.002; *R*^2^ = 0.37, [0.700–3.11]; *M* = 3.51) than when the recipient was a high social class adolescent (*M* = 3.38). Furthermore, empathy positively predicted caring tendencies in general (*β* = 0.41, *p* = 0.002, *R*^2^ = 0.37, [0.150–0.633]). In addition, we found an interaction between the recipient’s social class and empathy (*β* = −1.45, *p* = 0.002, *R*^2^ = 0.37, [−0.905 to −0.211]) that was qualified by a three-way interaction of Recipient’s social class × Participant’s social class × Empathy (*β* = 1.45, *p* = 0.007¸ *R*^2^ = 0.37, [0.184–1.12]). To disentangle this interaction, we ran simple slopes analysis. We found that for low-class participants, empathy had a positive effect on caring tendencies, but only in the condition where the recipient of help belonged to the low-class group (*β* = 0.40, *p* = 0.002), and not for the high-class recipient condition (*β* = −0.17, *p* = 0.20). Interestingly, we found the reverse pattern for high-class participants: empathy had no effect under low-class recipient condition (*β* = 0.18, *p* = 0.16), but showed a positive effect when the recipient of help belonged to the high-class group (*β* = 0.29, *p* = 0.02; Figure 2). In other words, empathy had a positive effect on caring tendencies for both low and high-class participants, but this effect was only true when the recipient of help belonged to the participants’ ingroup. When the recipient of help belonged to a different social group, empathy did not affect participants’ caring tendencies toward others. The dataset supporting these results is available under request.

## Discussion

Prosocial behavior represents a protective factor and is framed as an antecedent of social cohesion (Luengo Kanacri and Jiménez-Moya, 2017). For this reason among others, during the last decades, many researchers have focused on prosociality. Special attention has been paid to the relation between prosociality and social class, given that this social category is extremely determinant in current societies characterized by social inequality. However, this relation has not been yet resolved. In this work, we aimed to shed light on the controversial relation between social class and prosociality by including three aspects that might help understanding the contradictory results on this matter, namely the role of empathy and the role of the social class of the recipient of prosociality, and the consideration of different types of prosocial behavior. Furthermore, we featured an adolescent sample, given that prosocial behavior is especially relevant at this stage (Eisenberg et al., 2006).

Results showed that empathy had a positive effect on support for helping, but only for high-class participants. In this case, an increase in empathy is related to an increase in support for helping others, regardless of the social class of the recipient of help. However, in the case of low-class adolescents, empathy showed no effect on support for helping. Thus, based on our results, empathy does not affect helping tendencies for low-class individuals. On the contrary, empathy seems to be a relevant factor that might increase helping behavior among high-class individuals. This is especially interesting given that low social class individuals are, in general, more empathetic and accurate than high-class individuals, at least when it comes to judge others’ emotions (Kraus et al., 2010). The fact that empathy had different effects for high and low-class participants might respond to distinct motivations for each group. This way, even though low-class individuals tend to show higher levels of empathy, this seems not related to prosociality.

With regard to caring tendencies, we found that participants reported higher levels of caring when the recipient of help was a low-class adolescent, in line with research showing that individuals consider wealth information when it comes to prosociality (Sigelman and Waitzman, 1991; Paulus, 2014). More interestingly, and in line with our hypothesis, we show that empathy had a positive effect on caring tendencies, but only when the recipient of help was an ingroup member. For high social class participants in the condition where the recipient was also a high-class individual, empathy was related to an increase of caring tendencies. This effect was not true when the recipient of help belonged to the low-class group. We found the same pattern for low-class participants: Empathy had a positive effect on caring tendencies only when it was directed at other low-class adolescents. Therefore, we could say that both low and high social class participants showed an ingroup bias (Dovidio et al., 2017). This is contrary to previous results showing that “fairness” (to give more to the ones who need it) seems to be more relevant than “similarity” (to give more to other ingroup members) when it comes to prosociality (Van Doesum et al., 2017). However, as we mentioned earlier, note that the Chilean society is extremely unequal and social class differences are much more explicit and have stronger consequences, compared to other countries. In such a segregated context, ingroup membership might be a more relevant factor when it comes to caring for others.

More research is needed to confirm these tendencies in other contexts. However, we argue that these results might be enlightening in two ways. First, they show that in order to disentangle the relation between social class and prosocial behavior, it is relevant to take into account the social class of the recipient of help. According to our results, this might be especially pertinent when it comes to caring, as we found that empathy has a positive effect only when the recipient of help belongs to the same social group. The fact that this is not happening with helping might be related to the notion that different types of prosocial behaviors may have diverse routes and antecedents (see Eisenberg et al., 2006) and imply different levels of involvement. Our conceptualization of helping implies fewer costs for the helper than caring behavior, which is related with stronger ties and a high involvement. Our results show that empathy is related to high levels of prosociality when the recipient of help is an ingroup member and when the type of prosociality implies a high cost. According to this, the relation between empathy or sympathy and prosociality is more consistent in the case of relatively costly prosocial acts (Eisenberg et al., 2002). However, more research is needed to explore the potential distinctions among different forms in which prosociality is enacted (for instance, prosocial risk taking, see Do et al., 2017) and its relation with social class.

Second, these results seem to confirm the fact that empathy is not necessarily useful for improving cooperative social interactions (see Stürmer et al., 2005), since under certain conditions, its positive effect only benefits the ingroup, maintaining prejudiced attitudes and acts toward the ones who are different (see Bloom, 2016). However, this should be taken with caution, given that other factors might also affect prosocial behavior.

Our results do not answer the well-known question of who helps more, whether those who have less or those who hold a higher status. However, we show that the interplay of social class of the helper and the recipient of help, and empathy, are useful to better understand the relation between social class and different types of prosociality. In other words, in line with recent discussions, we highlight the fact that both personality and social psychology perspectives are needed to understand the social phenomenon of inter-group helping between high and low social class individuals (see Matthews, 2020).

Although we used genuinely low and high social class groups, future studies should measure actual prosocial behavior in different contexts. This would allow us to better understand the extent to which social class is a relevant factor when it comes to prosocial behavior. Besides, the results presented in this work should be extended and applied to other social groups. The distinct effects of empathy might not be unique to high and low social class individuals, but applicable to other groups with similar power asymmetries.

In conclusion, future studies are needed to disentangle the complex relation between social class and prosocial behavior. However, we have shown that at least empathy and the social class of the recipient of help seem to be two factors that contribute to understand this challenging relation.

## Data Availability Statement

The data that support the findings of this study are available from the corresponding author upon request.

## Ethics Statement

The studies involving human participants were reviewed and approved by Comité Científico de Ciencias Sociales, Artes y Humanidades, Pontificia Universidad Católica de Chile. Written informed consent to participate in this study was provided by the participants’ legal guardian/next of kin.

## Author Contributions

GJ-M, BL, PC, and MM contributed to conception and design of the study. GJ-M implemented the study and organized the database. GJ-M and BL performed the statistical analysis. GJ-M wrote the first draft of the manuscript. CB wrote sections of the manuscript. All authors contributed to manuscript revision, read, and approved the submitted version.

### Conflict of Interest

The authors declare that the research was conducted in the absence of any commercial or financial relationships that could be construed as a potential conflict of interest.
